# Work-Related Quality of Life in Tunisian Medical Interns: Key Influencing Factors

**DOI:** 10.1192/j.eurpsy.2025.1587

**Published:** 2025-08-26

**Authors:** M. Hajji, N. Rmadi, A. Hrairi, N. Kotti, M. Hajjaji, K. Jmal Hammami

**Affiliations:** 1 Family medicine department, university of Sfax; 2 Occupational Medicine department, Hedi Chaker university hospital, University of Sfax, Sfax, Tunisia

## Abstract

**Introduction:**

Work-related quality of life (WRQoL) is a crucial aspect of overall well-being, particularly for medical interns who face unique challenges in their demanding environment.

**Objectives:**

This study aims to assess factors associated to work-related quality of life in a population of Tunisian Medical Interns.

**Methods:**

A cross-sectional study was conducted from July to September 2024 among Tunisian medical interns using a Google Form’s questionnaire. They were asked about their sociodemographic characteristics and their working conditions such as number of working hours per day, number of night shifts per month, and number of patients seen per day. Quality of working life was measured using the the Work-Related Quality of Life (WRQoL) Scale which covered six domains: General Well-Being (GWB), Home-Work Interface (HWI), Job and Career Satisfaction (JCS), Control at Work (CAW), Working Conditions (WCS) and Stress at Work (SAW).

**Results:**

Our study included 141 interns with a mean age of 27.28 ± 2.42 years. Among them, 31 (22.1%) were married. The average working hours per day was 6 .42 hours, and the average number of patients seen per day was 12 patients. The average nightshifts done per month was 6. Age was statistically positively associated with all WRQoL’ domains. However, GWB and HWI were negatively associated with number of working hours per day (p=0.022 and 0.026 respectively) and number of night shifts per month (p=0.001 and 0.000 respectively). Moreover, JCS, CAW and WCS were negatively associated with number of night shifts per month (p=0.007, 0.002 and 0.000 respectively).

**Conclusions:**

This study highlights the significant factors influencing work-related quality of life among Tunisian medical interns such as working conditions. By addressing these factors, it is possible to enhance the overall quality of life for medical interns, thereby promoting their well-being and improving their capacity to provide quality patient care.

**Disclosure of Interest:**

None Declared

